# The encounter

**Published:** 2019-03-13

**Authors:** Nova Zhao

**Affiliations:** 1 McMaster University, Ontario, Canada

## Abstract

We know that homelessness is a nation-wide problem in Canada. What may be often forgotten, especially by those of us unfamiliar with poverty and deprivation, is how homelessness affects a person’s health. In this essay, I narrate a chance encounter with a homeless man that taught me the difficulties homeless people face in accessing, navigating, and benefiting from the health care system. We need to rethink how we health care providers respond to the homeless, and medical students can help lead the change.

Every day on my walk to and from school, I pass through a lovely public park. Usually, I walk at a fairly quick pace so as not to waste time or risk being late for class, ignoring those around me and deliberately avoiding eye contact. One afternoon on my brisk walk through the park, a dishevelled-looking man on a bench asked me for money. On most days, I would pretend I didn’t hear him or make a lame excuse like “I don’t carry change.” But on this particular day, I decided to chat with the man and listen to his story. He told me his name was J* and that he was a missionary from Vancouver. When I asked J what brought him to Kitchener, he revealed that he travelled here to see his mother, who had recently suffered a stroke and was very frail. Nonetheless, upon arriving, with no income or money, he found himself begging and sleeping in shelters.

We then chatted about astronomy, medical specialties (by this point I had already told J that I was a medical student), and nutrition. During our conversation, I glanced down at his legs and noticed what seemed like severe cellulitis. He admitted that he had contracted MRSA due to his poor living conditions and was unable to pay for the medications he was prescribed. That’s when the switch flipped and I understood his plight and that of many others like him. Prior to this encounter, I seldom stopped to consider the health of people I saw on the street or the implications of their living conditions. But now I realize that this population is largely neglected and underserved in Canada. After a quick search on the internet, I learned that homeless men in Toronto have a mortality rate three times higher than the mean.^1^ Also, homeless adults often develop certain conditions much earlier than non-homeless populations.^2^ They suffer from preventable illness and death because of factors often out of their control, such as extreme poverty, cognitive impairment, and difficulties accessing health care. The problem is worse in some cities; in the Waterloo Region alone, there were almost three thousand people who experienced homelessness in 2017.^3^ Three thousand people at risk of diseases and illnesses last year that most of us can easily avoid by virtue of our clean homes and running water. This made me ask myself: what am I doing to help?

I gave J some change so he could buy himself a meal, and a few cough drops to relieve his sore throat. However, this issue is much more complex than just food and comfort. In order to make change, we need to address everything from basic necessities like food and housing, to community-level programs, government and healthcare policy, and societal beliefs. Unfortunately, I don’t possess the knowledge or power to effect change in all of these domains, but what I *can* do, as a medical student and future physician, is advocate. I can advocate for J and others like him who are underserved in our healthcare system. That’s why I wrote this piece - to expose the difficulties that homeless individuals experience in health care and to advocate for change amongst my peers and fellow health professionals.

Change can be as simple or as involved as you want it to be. One area where medical students can directly effect change is student-run clinics (SRCs). There are several SRCs across Canada that are trying to address the healthcare needs of the homeless population. For example, The University of Calgary Student Run Clinic, founded in 2010, delivers primary care services at multiple inner city sites.^4^ It has played a significant role in improving access to primary care for Calgary’s homeless population as well as educating medical students on the importance of serving marginalized populations.^5^ Saskatchewan also has two SRCs; SWITCH (Student Wellness Initiative Towards Community Health)^6^ and SEARCH (Student Energy in Action for Regina Community Health),^7^ both of which provide equitable healthcare services to inner city populations in Saskatoon and Regina, respectively.

Another way to be an agent of change is by advocating for equitable access to healthcare, particularly for homeless individuals, at events such as the CFMS Lobby Day. You can also write to your local MP about what the government is doing to help underserved populations in your community. Advocacy is, after all, one of the principal methods of overcoming barriers in healthcare on a large scale.

But first and foremost, I invite my peers to start by questioning their own beliefs and behaviours towards homeless people. I know that from now on, whenever I encounter a homeless individual, I will make an effort to challenge my own attitudes and stop to chat with them. You can’t fully understand what someone else might be going through unless you hear their story.

## Footnotes

* Name changed for privacy.
